# Investigating the Prospective Sense of Agency: Effects of Processing Fluency, Stimulus Ambiguity, and Response Conflict

**DOI:** 10.3389/fpsyg.2017.00545

**Published:** 2017-04-13

**Authors:** Nura Sidarus, Matti Vuorre, Janet Metcalfe, Patrick Haggard

**Affiliations:** ^1^Institute of Cognitive Neuroscience, University College LondonLondon, UK; ^2^Institut Jean Nicod, Département d’Etudes Cognitives, ENS, EHESS, CNRS, PSL Research UniversityParis, France; ^3^Department of Psychology, Columbia University, New YorkNY, USA

**Keywords:** sense of agency, action selection, motor control, metacognition, fluency

## Abstract

How do we know how much control we have over our environment? The sense of agency refers to the feeling that we are in control of our actions, and that, through them, we can control our external environment. Thus, agency clearly involves matching intentions, actions, and outcomes. The present studies investigated the possibility that processes of action selection, i.e., choosing *what* action to make, contribute to the sense of agency. Since selection of action necessarily precedes execution of action, such effects must be prospective. In contrast, most literature on sense of agency has focussed on the retrospective computation whether an outcome fits the action performed or intended. This hypothesis was tested in an ecologically rich, dynamic task based on a computer game. Across three experiments, we manipulated three different aspects of action selection processing: visual processing fluency, categorization ambiguity, and response conflict. Additionally, we measured the relative contributions of prospective, action selection-based cues, and retrospective, outcome-based cues to the sense of agency. Manipulations of action selection were orthogonally combined with discrepancy of visual feedback of action. Fluency of action selection had a small but reliable effect on the sense of agency. Additionally, as expected, sense of agency was strongly reduced when visual feedback was discrepant with the action performed. The effects of discrepant feedback were larger than the effects of action selection fluency, and sometimes suppressed them. The sense of agency is highly sensitive to disruptions of action-outcome relations. However, when motor control is successful, and action-outcome relations are as predicted, fluency or dysfluency of action selection provides an important prospective cue to the sense of agency.

## Introduction

As we interact with the world around us, our experience is typically colored by a sense of agency, a feeling that we are in control of our actions and, through them, can control events in the outside world (Haggard and Tsakiris, 2009). The human sense of agency is a critical part of our subjective experience, and serves to ground our sense of self (Knoblich et al., 2003). Its importance is further highlighted in several pathologies that involve disorders of the sense of agency, such as schizophrenia, obsessive-compulsive disorder, or alien-hand syndrome (Moore and Fletcher, 2012). Finally, our experience of agency also underlies our societal notions of responsibility, and “free will,” on which our legal system is based (Spence, 2009).

Much research has focused on how actions are linked to their outcomes. This has shown that the sense of agency depends on a retrospective comparison between expected or desired action outcomes and actual outcomes (e.g., Wegner and Wheatley, 1999; Blakemore et al., 2002). Moreover, the importance of linking intentions and actions has been recently highlighted (for a review, see Chambon et al., 2014b). In fact, modern theoretical frameworks emphasize that the sense of agency results from the integration of multiple cues (Synofzik et al., 2008; Moore and Fletcher, 2012), which may become available at different times (Haggard and Chambon, 2012; Farrer et al., 2013). Moreover, metacognitive processes are involved in evaluating the output of action and outcome monitoring systems (Metcalfe and Greene, 2007; Haggard and Chambon, 2012; but see Chambon et al., 2014a).

Recent studies have used subliminal priming of actions to manipulate the fluency of action selection, in a simple paradigm in which participants respond according to directional arrows and trigger the appearance of colored circles (Wenke et al., 2010; Chambon and Haggard, 2012; Chambon et al., 2013, 2015; Sidarus et al., 2013). Participants report a reduced sense of agency over action outcomes when primes induce dysfluent, compared to fluent, action selection. Similar findings have also been seen with response conflict induced by supraliminal stimuli, i.e., incongruent flankers (Sidarus and Haggard, 2016). Therefore, a metacognitive signal about the (dys)fluency of action selection processes contributes to the sense of agency prospectively, and long before the outcome is known.

Interestingly, it has been shown that judgements of agency (JoAs) are influenced by the metacognitive monitoring of performance in a game, but are still highly sensitive to *actual* disruptions of control (Metcalfe and Greene, 2007). In these studies, a computer game was used in which participants move a mouse cursor (a box) along a horizontal bar to catch falling Xs, while avoiding Os. Participant’s motor actions produce two levels of outcome. First, moving the mouse leads to the cursor moving on the screen: this may be considered the *proximal* outcome. Introducing a discrepancy between one’s mouse movements and cursor movements (termed “turbulence”) leads to a reduction in both performance and judgements of performance (JoPs), but an even greater reduction in JoAs. Importantly, multiple regression models confirmed that the large reduction in JoAs due to discrepant feedback could not be fully explained by reductions in performance. In the same task, a second, *distal* level of outcome occurs. The participant’s goal is to catch Xs, thus making them disappear, and the motion of the visual cursor is the means whereby they achieve this goal. In trials in which Xs could sometimes disappear autonomously, participants were aware of the corresponding improvement in their performance, but reported a reduced sense of agency compared to trials where Xs could only disappear because they participant caught them. Notably, the reduction in JoAs due to discrepant feedback was much greater than the reduction associated with Xs disappearing unexpectedly. That is, the sense of agency is more sensitive to disruption of proximal outcomes, compared to distal outcomes (cf. Metcalfe et al., 2013). Together, these results suggest that the sense of agency is preferentially tuned to monitor proximal cues, tied to action and motor control.

Here we introduce a new potential cue to sense of agency in the same task, by varying the difficulty of action selection. In this task, we may consider that an action consists of moving the cursor to the horizontal location of a target (i.e., an X). This means action selection would be determined by: first, detecting a stimulus; second, categorizing it as a target or distractor; and, third, deciding whether to move the cursor toward it (for targets), while also avoiding distractor stimuli (i.e., O’s). Therefore, with some changes to the stimuli, we could manipulate these three different stages of action selection.

This allowed us to test the generalisability of the effects of action selection across manipulations, but also in a dynamic environment, with greater ecological validity than the experimental paradigms typically used to study the sense of agency. In goal-directed action, the sense of agency depends on both prospective factors, and on proximal outcomes that constitute the means for achieving the goal, in addition to the actual goal itself. Therefore, we additionally aimed to compare the relative contribution to sense of agency of both prospective cues and proximal outcomes, for the same task, and the same distal goal. Manipulations of action selection were thus combined with manipulating the relation between one’s movement and the visual cursor used to catch the Xs.

Many studies have shown that fluency in visual processing or decision-making can affect a variety of judgements, such as confidence, liking, or familiarity (for a review, see Alter and Oppenheimer, 2009). Previous studies, which used action priming, argued that action selection fluency influences the sense of agency (e.g., Wenke et al., 2010). Priming can influence processing fluency, in addition to inducing response conflict, but it remains unclear whether fluency in perceptual processing alone could have a similar effect on the sense of agency. To test this, in Experiment 1, we manipulated stimulus processing fluency through visual masking. This essentially disrupted the very first stage of the stimulus-response-outcome chain, namely, identifying and locating target stimuli. If fluency in action selection has a general effect on the sense of agency, we would predict that this manipulation would lead to a reduced sense of agency.

In Experiment 2, we manipulated the uncertainty associated with categorizing stimuli as targets or distractors by varying stimulus ambiguity. When categorizing highly ambiguous stimuli, uncertainty about the accuracy of the categorization would be higher. It has been suggested that the consequences of uncertain decisions may be seen as less blame-worthy than the consequences of more informed decisions (Heath and Tversky, 1991). Thus, greater uncertainty during action selection could lead to a reduction in the sense of agency. Additionally, a large number of highly ambiguous stimuli would render action selection processes more difficult, as many items would be hard to categorize. This increased difficulty could also lead to a lower sense of agency.

Finally, in Experiment 3, we interfered with the later, pre-motor aspects of action selection, namely, deciding whether to approach or avoid a given stimulus. Response conflict was induced by placing incongruent flankers around central targets or distractors (Eriksen and Eriksen, 1974). For example, M could indicate targets and C indicate distractors. These items would appear surrounded by congruent (e.g., MMM) or incongruent (e.g., CMC) flankers. The simultaneous detection of a target and a distractor would suggest two conflicting responses: approach and avoid that spatial location. Detecting a distractor flanker could elicit an avoidant response, which would need to be overcome if it was flanking a target; while an approach response could be erroneously elicited by a target flanker placed around a distractor. A condition in which only congruently flanked items appeared was compared to a condition in which most items were congruently flanked, and to a condition in which few items were congruently flanked. The overall degree of response conflict was expected to lead to a corresponding reduction in the sense of agency.

## Experiment 1

This experiment investigated the effects of visual processing fluency on the sense of agency, by adding a visual noise mask on the screen in some trials.

### Materials and Methods

#### Participants

Twenty-three Columbia University or Barnard College students volunteered to participate for course credit, and gave written informed consent (12 female, mean age = 20.05, *SD* = 2.52, age not recorded for 1 person due to technical error). All were right-handed, with normal or corrected-to-normal vision, and neurologically healthy. The procedures described here conform to the guidelines of the APA concerning the protection of human subjects, and were approved by the Columbia Internal Review Board.

#### Apparatus

The experiments were conducted on iMac computers, using a mouse on mouse pad. The program was developed using custom-built scripts running on Python. White Xs and Os were presented on a gray background. Each trial started with 10 stimuli of each type (targets vs. distractors). In unmasked trials, the background was gray (115/255 RGB scale). In masked trials, the noise mask consisted of a Gaussian-filtered patch of randomly distributed grayscale intensities. This was applied on top of the main game screen, except for the half gray horizontal bar and white box (i.e., the mouse cursor), which were drawn on top of the mask. This enabled masking of the stimuli based on which participants decided *what* to do, while allowing participants to track their movements equally well in both masked and unmasked conditions. On some trials, we also introduced a discrepancy between the participant’s movements of the mouse, and the movements of the cursor on the screen, termed “turbulence.” In the turbulence condition, the movement of the box depended on the following noise function:

Δx'=Δx+σsin(2πt/2.4)

where Δ*x′* is the movement of the box on the screen, Δ*x* is the distance the participant actually moved the mouse, *t* is time in seconds, and *σ* is the amplitude of the noise wave.

#### Design and Procedure

**FIGURE 1:**
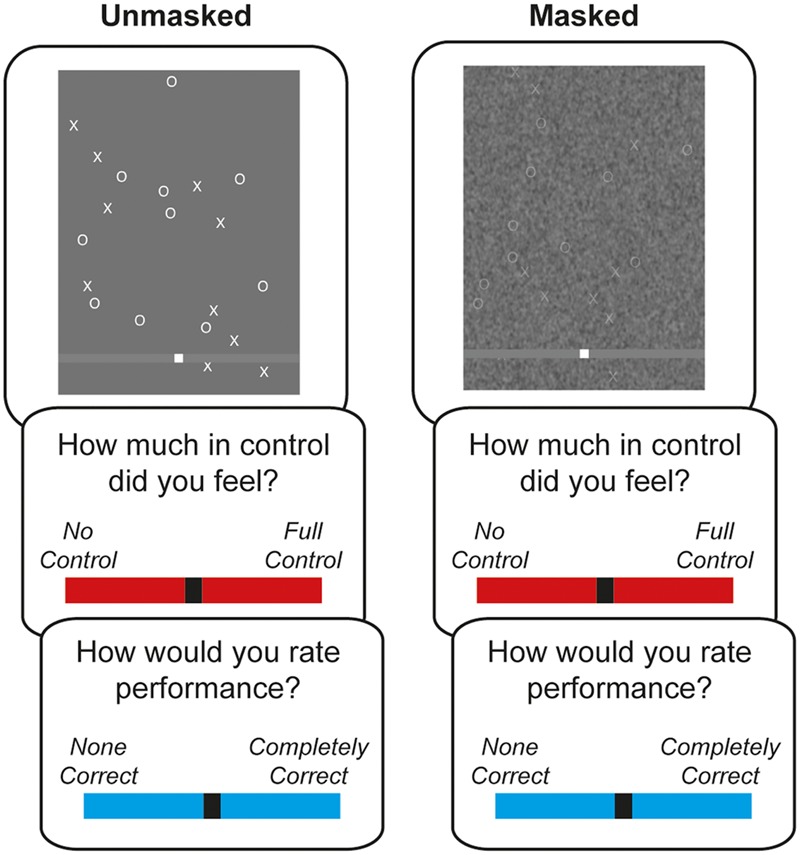
**Task outline for Experiment 1.** Visual processing fluency was manipulated by adding a visual noise mask on some trials. After playing the game for 30 s, participants gave judgements of agency (on a red VAS), followed by judgements of performance (blue VAS).

The basic procedure and instructions are described elsewhere (Metcalfe and Greene, 2007). Briefly, participants played a game in which they observed Xs and Os scrolling down a screen, and moved a white box along a gray horizontal track with a mouse (see **Figure 1**). Participants were instructed to catch one letter with their white box (e.g., Xs), while avoiding the other letter (e.g., Os; counterbalanced between participants). Once caught, the items disappeared, and auditory feedback indicated whether a target or distractor was hit, with a ping or thud sound, respectively. If the items were not caught, they continued scrolling down to the bottom of the screen.

Participants played the game for 30 s, and then gave JoAs and JoPs about the game they had just played. For the JoAs, participants were asked to judge how much control they felt over the game, using a red visual analog scale (VAS) ranging from “No Control” to “Full Control.” For JoPs, participants were asked to rate their performance in the game using a blue VAS, ranging from “None Correct” to “Completely Correct.” Participants moved a slider with the mouse, and pressed the space bar to select their rating.

To influence action selection, the fluency of visual processing was manipulated by either presenting a normal screen (“unmasked” trials), or adding a visual noise mask on top of the game (“masked” trials; see **Figure 1**). This masking made it harder to detect the targets and distractors, thus rendering action selection more difficult. Additionally, to interfere with proximal outcome monitoring, the movements of the mouse cursor (the white box) were manipulated. In some trials, the cursor accurately followed the movement of the mouse (“no turbulence”). In other trials, a noise function was applied to the movements of the cursor (“turbulence”). These two manipulations, visual masking and cursor turbulence, were factorially combined, resulting in four trial (i.e., game) types. These were quasi-randomized across six blocks, such that each trial type was played once before the next block.

Before starting the experiment, participants played a training game, followed by JoAs and JoPs. They were given a chance to ask any questions, and either play another training game, or start the experiment. At the end of the experiment, participants answered a short questionnaire about the experiment and were debriefed.

#### Data Analysis

Performance in the game was assessed as a *d’* score from signal detection theory (Green and Swets, 1966), which measured discrimination between targets and distractors. The *d’* calculation was adjusted for instances of zero false alarms (Snodgrass and Corwin, 1988; Mill and O’Connor, 2014). JoAs and JoPs were quantified as a percentage of the VAS scale. Mean *d’*, JoAs and JoPs were submitted to repeated measures ANOVAs, with the factors masking (unmasked vs. masked) and turbulence (no turbulence vs. turbulence).

Additionally, we assessed whether any effects of visual masking on JoAs could be explained by a reduction in JoPs. For this, a hierarchical linear regression model (also known as linear mixed-effects models) was used to model single-trial level data. JoAs were modeled by the factors masking and turbulence (coded as unmasked = 0, masked = 1; no turbulence = 0, turbulence = 1), as well as their interaction. JoPs were added as a covariate, after standardizing within participants, as we predicted that JoPs would inform JoAs across conditions. All fixed effects were also allowed vary between participants (i.e., participant random intercepts and slopes). This analysis was conducted using the *lme4* package (Bates et al., 2014) in R (R Core Team, 2015). Parameter estimates (*b*) and their associated *t*-tests (*t, p*), calculated using the Satterthwaite approximation for degrees of freedom (Kuznetsova et al., 2015), are presented to show the magnitude of the effects, with bootstrapped 95% confidence intervals.

### Results

**FIGURE 2:**
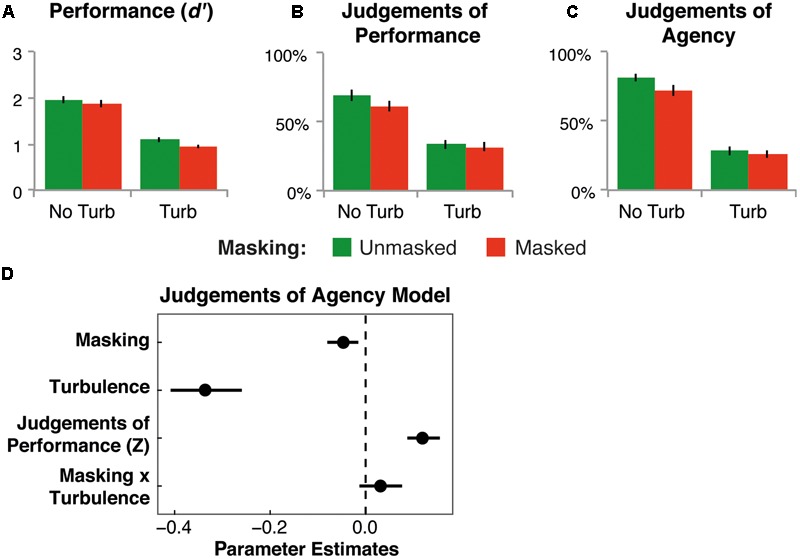
**Results of Experiment 1.** Effects of masking and turbulence on mean *d*’ **(A)**, JoPs **(B)**, and JoAs **(C)**. No Turb, no turbulence; Turb, turbulence. Error bars show the standard error of the mean. **(D)** Parameter estimates, with bootstrapped 95% confidence intervals, for modeling JoAs by the factors masking and turbulence, and by JoPs (within-participants *Z*-score).

Analysis of *d’* showed that discrimination between targets and distractors was significantly lower for masked, relative to unmasked, trials [mean difference = 0.12, *SD* = 0.15, *F*_(1,22)_ = 13.67, *p* = 0.001, $\eta_{p}^{2}$ = 0.38; see **Figure 2A**]. Turbulence also led to a significant reduction in *d’*, relative to no turbulence trials [mean difference = 0.89, *SD* = 0.25, *F*_(1,22)_ = 302.66, *p* < 0.001, $\eta_{p}^{2}$ = 0.93]. There was no significant interaction between the factors [*F*_(1,22)_ = 0.91, *p* = 0.35, $\eta_{p}^{2}$ = 0.040].

Similarly, JoPs were significantly lower for masked, relative to unmasked, trials [mean difference = 4.60%, *SD* = 4.73, *F*_(1,22)_ = 20.66, *p* < 0.001, $\eta_{p}^{2}$ = 0.48; see **Figure 2B**]. Turbulence also led to a significant reduction in JoPs, relative to no turbulence trials [mean difference = 32.67%, *SD* = 17.96, *F*_(1,22)_ = 75.83, *p* < 0.001, $\eta_{p}^{2}$ = 0.78]. There was a marginally significant interaction between the factors [*F*_(1,22)_ = 3.53, *p* = 0.074, $\eta_{p}^{2}$ = 0.14].

Results for JoAs (see **Figure 2C**) showed a significant reduction for masked, relative to unmasked, trials [mean difference = 6.13%, *SD* = 5.39, *F*_(1,22)_ = 28.52, *p* < 0.001, $\eta_{p}^{2}$ = 0.57]. Turbulence also led to a significant reduction in JoAs, relative to no turbulence trials [mean difference = 49.58%, *SD* = 18.67, *F*_(1,22)_ = 161.65, *p* < 0.001, $\eta_{p}^{2}$ = 0.88]. Moreover, there was a significant interaction between the factors [*F*_(1,22)_ = 5.81, *p* = 0.025, $\eta_{p}^{2}$ = 0.21]. Simple effects *t*-tests showed that, for no turbulence trials, masking led to a significant reduction in JoAs [mean difference = 9.48%, *SD* = 10.65, *t*_(22)_ = 4.27, *p* < 0.001, *d*_z_ = 0.89]; whereas this reduction was only marginally significant for turbulence trials [mean difference = 2.65%, *SD* = 6.19, *t*_(22)_ = 2.05, *p* = 0.053, *d*_z_ = 0.43]. Turbulence had a significant effect in both masking conditions (unmasked: mean difference = 52.97%, *SD* = 19.91, *t*_(22)_ = 12.76, *p* < 0.001, *d*_z_ = 2.66; masked: mean difference = 46.14%, *SD* = 19.87, *t*_(22)_ = 11.14, *p* < 0.001, *d*_z_ = 2.32).

Finally, we assessed whether the effect of masking on JoAs could be accounted for by changes in JoPs. Previous studies (e.g., Metcalfe et al., 2013) showed that perceived performance is used as cue to agency, predicting a general, positive relation between JoPs and JoAs. Therefore, JoAs were modeled by the experimental factors, and JoPs (standardized) were entered as a covariate (see **Figure 2D** and Supplementary Table 1). The results revealed that JoPs were positively related to JoAs (*b* = 0.12, *t*_(22.56)_ = 6.39, *p* < 0.001, 95% CI = [0.09, 0.16]), as predicted. Importantly, masking remained a significant predictor of JoAs (*b* = -0.05, *t*_(21.53)_= -2.98, *p* = 0.007, 95% CI = [-0.08, -0.02]), as did turbulence (*b* = -0.34, *t*_(20.27)_= -8.80, *p* < 0.001, 95% CI = [-0.41, -0.26]). The interaction between masking and turbulence was no longer significant (*b* = 0.03, *t*_(51.91)_ = 1.48, *p* = 0.14, 95% CI = [-0.01, 0.08]).

### Discussion

In Experiment 1, visual masking was used to disrupt the processing fluency of the stimuli that drove participants’ actions. This, in turn, disrupted the fluency of action selection, as it made the process of deciding which action to make, i.e., where to move the cursor, more difficult. The results showed that visual masking disrupted both objective and subjective measures of performance, as expected. Moreover, visual masking led to a reduction in JoAs, which could not be explained by perceived differences in performance, due to increased task difficulty. That is, the mere disruption of processing fluency led to a loss of agency.

These results are consistent with research showing that fluency can affect a number of metacognitive judgements (see Alter and Oppenheimer, 2009 for a review). Moreover, previous studies have shown that dysfluent action selection is associated with a reduction in the sense of agency (Chambon et al., 2014b). These studies used response conflict to manipulate the fluency of action selection. Here, we show that disrupting action selection at the early stage of stimulus processing can lead to a reduction in the sense of agency.

In addition to visual processing, we manipulated whether the consequences of one’s actions matched one’s intentions, by introducing a discrepancy between the movement of the mouse and the movement of the cursor, termed turbulence. When the cursor on the screen did not accurately track the mouse’s movements, there was a reduction in both objective and subjective measures of performance, as well as a large reduction in JoAs. This reduction in JoAs was independent of differences in JoPs, and was larger than the effect of turbulence on JoPs. These findings replicate previous studies that used a similar manipulation (e.g., Metcalfe and Greene, 2007; Metcalfe et al., 2013).

Finally, while discrimination performance reflected additive effects of the visual masking and mouse turbulence manipulations, the effects of these factors on metacognitive judgements of agency were partially underadditive. This underadditivity can be seen in the significant interaction between visual masking and turbulence effects on average JoAs. Overall, visual masking had a minor effect on JoAs compared to the effect of turbulence. Additionally, we found that visual processing dysfluency especially disrupted the sense of agency when the cursor accurately followed the movements of the mouse, but less so when turbulence was introduced. On the other hand, turbulence had a large and robust effect across masking conditions. The larger effect of turbulence on performance suggests that it may have been a more salient cue for agency than visual processing fluency. The greater saliency of discrepant action feedback resulted in very low JoAs, i.e., a floor effect, which in turn obscured the effects of processing fluency on JoAs observed under accurate action feedback. In fact, average JoPs also showed a somewhat underadditive effect of the two manipulations, possibly denoting that turbulence was a stronger cue for both metacognitive judgements.

Nonetheless, the interaction between masking and turbulence on JoAs was no longer significant after accounting for differences in JoPs, in a multi-level regression model. We may speculate that the apparent underadditive effect is due to the difference in the size of the effects of masking and turbulence on JoAs. Future studies should attempt to balance the effects of masking and turbulence on performance, to clarify whether these underadditive effects on metacognitive judgements are linked to differences in the salience of the two manipulations. One may speculate that they are not, given that mouse turbulence is a reliable and direct indicator of a loss of agency. Participants were less able to implement their intended actions, and thus had objectively less control over the game. Visual masking only made action selection more difficult, but did not interfere with the ability to interact with the game. Alternatively, selection fluency may have a general effect on JoAs, consistent with other work on the effects fluency on metacognitive and affective judgements (Alter and Oppenheimer, 2009).

## Experiment 2

This experiment aimed to investigate whether the ease, or difficulty, of classifying stimuli as targets for action would influence sense of agency, by varying stimulus ambiguity.

### Materials and Methods

#### Participants

Procedures were as described above. Twenty-four new Columbia University or Barnard College students (10 female, mean age = 20.79, *SD* = 2.93) volunteered to participate for course credit, and gave written informed consent. Five participants were left-handed, and the remaining were right-handed.

#### Apparatus

The basic setup was as in Experiment 1, except for the following. The target and distractor items now consisted of 6 gray circles, of 3 lighter and 3 darker shades relative to half gray (roughly 65, 90, 115, 140, 165, and 190 on a 255 RGB scale). The horizontal bar was now white, and the box controlled by the participant was black. The number of items at the start of the trial was reduced to six targets and six distractors, in order to increase overall performance. The turbulence manipulation was adjusted to double the period of the sine wave (by dividing 2π *t* by 4.8, instead of 2.4). This slowed down the rate at which the direction of the noise component added to the mouse’s movement changed, thus making the mouse more controllable than in Experiment 1.

#### Design and Procedure

**FIGURE 3:**
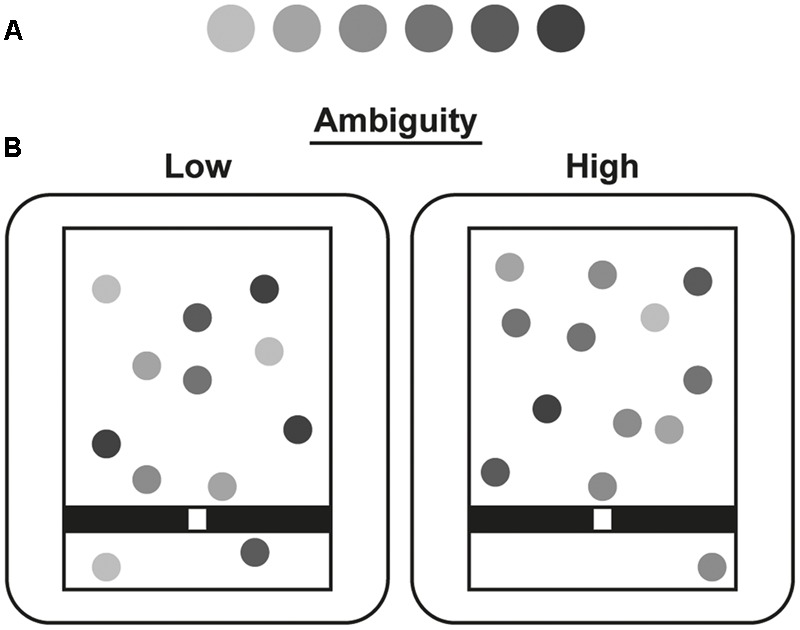
**Schematic of stimuli in Experiment 2. (A)** The six graded shades of gray used. The first three stimuli were classed as light gray, whereas the last three were classed as dark gray. **(B)** The two ambiguity conditions, which differed in their proportions of extreme and ambiguous gray shades. Stimulus size and colors have been adapted.

The main design and procedures were as in previous experiments. Instead of letters, the items consisted of gray circles. Participants were instructed to catch all the light gray circles, i.e., the targets, but to avoid the dark gray circles, i.e., the distractors (counterbalanced). Here, action selection was manipulated by varying the ambiguity in categorizing items as targets or distractors. There were six equally spaced shades of gray, bisected by the half gray tone (see **Figure 3A**). Thus, there were three levels of ambiguity in the gray shades, depending on the distance from half gray. The two shades at the extremes of the range were easy to categorize as light or dark, whereas the two shades closest to half gray were highly ambiguous.

Ambiguity was manipulated across trials (i.e., games), by varying the proportions of more and less ambiguous shades (see **Figure 3B**). In the low ambiguity condition, most items were drawn from the extremes of the range. Thus, each trial started with three extreme, two medium, and one very ambiguous shade, both for targets and distractors. In the high ambiguity condition, most of the items were drawn from the highly ambiguous end of the range. Each trial started with one extreme, two medium, and three very ambiguous shades of targets and of distractors. As before, turbulence was also manipulated across trials. As before, the four resulting conditions were quasi-randomized, across six blocks. Participants played the game for 30 s, and then gave JoAs, and JoPs.

During training, participants were shown the three shades of gray to class as targets, and the three shades to class as distractors. There were two practice games, both followed by JoAs and JoPs.

#### Data Analysis

Mean *d’*, JoPs and JoAs were submitted to repeated measures ANOVAs with the factors ambiguity (low vs. high) and turbulence (no turbulence vs. turbulence). Simple effects *t*-tests were used to probe interaction between ambiguity and turbulence.

To characterize participants’ strategies, the response bias (*c*) measure from signal detection theory (Green and Swets, 1966) was used, with a correction for zero false alarms (Snodgrass and Corwin, 1988; Mill and O’Connor, 2014). Zero reflects an unbiased criterion, negative values reflect a liberal criterion, and positive values denote a conservative criterion. Essentially, here, a larger positive criterion would be associated with a lower number of items caught (targets or distractors), i.e., more careful responding. This bias measure was computed for each trial, and then averaged for each participant (collapsing across conditions).

Similarly to the previous experiments, JoAs were modeled by the independent variables and by JoPs. Ambiguity was coded as low = 0, high = 1. Additionally, average bias (*c*) was included as a between-participant (mean centered) covariate, as was the interaction between ambiguity and average bias.

### Results

**FIGURE 4:**
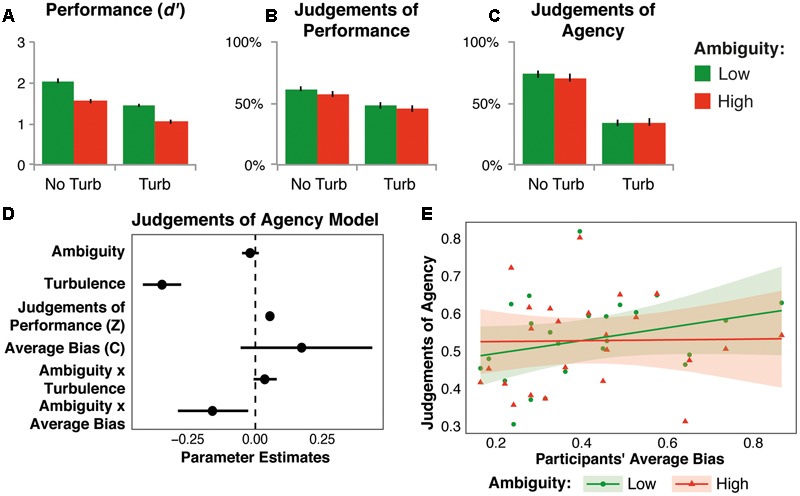
**Results of Experiment 2.** Effects of ambiguity and turbulence on mean *d’* **(A)**, JoPs **(B)**, and JoAs **(C)**. No Turb, no turbulence; Turb, turbulence. Error bars show the standard error of the mean. **(D)** Parameter estimates, with bootstrapped 95% confidence intervals, for modeling JoAs by the independent variables, JoPs (*Z*-score, within participants), and average bias (centered, between participants). **(E)** Average JoAs across participants (points) and model predictions (regression line, and shaded 95% prediction intervals) for the relation between the effect of stimulus ambiguity on JoAs and participants’ average response bias. Participants with a larger bias had a more conservative response criterion. Predictions were obtained from 10,000 simulations from the posterior distribution of plausible parameter values under uniform priors (Gelman and Su, 2015).

Discrimination between targets and distractors (*d’*) was significantly reduced by high ambiguity, relative to low ambiguity [mean difference = 0.45, *SD* = 0.20, *F*_(1,23)_ = 116.00, *p* < 0.001, $\eta_{p}^{2}$ = 0.84; see **Figure 4A**]. Turbulence also led to significantly lower d’ scores, relative to no turbulence [mean difference = 0.55, *SD* = 0.14, *F*_(1,23)_ = 362.92, *p* < 0.001, $\eta_{p}^{2}$ = 0.94]. There was no significant interaction between the factors [*F*_(1,23)_ = 1.57, *p* = 0.22, $\eta_{p}^{2}$ = 0.064].

High ambiguity led to a significant reduction in JoPs, relative to low ambiguity [mean difference = 3.04%, *SD* = 5.04, *F*_(1,23)_ = 8.73, *p* = 0.007, $\eta_{p}^{2}$ = 0.28; see **Figure 4B**]. Turbulence also led to significantly lower JoPs than no turbulence [mean difference = 12.77%, *SD* = 8.32, *F*_(1,23)_ = 56.62, *p* < 0.001, $\eta_{p}^{2}$ = 0.71]. There was no significant interaction between the factors [*F*_(1,23)_ = 1.51, *p* = 0.23, $\eta_{p}^{2}$ = 0.062].

Analysis of mean JoAs showed no significant main effect of ambiguity [*F*_(1,23)_ = 1.36, *p* = 0.26, $\eta_{p}^{2}$ = 0.056]. Turbulence led to lower significantly JoAs than no turbulence [mean difference = 37.91%, *SD* = 16.72, *F*_(1,23)_ = 123.33, *p* < 0.001, $\eta_{p}^{2}$ = 0.84]. Moreover, there was a significant interaction between ambiguity and turbulence [*F*_(1,23)_ = 5.55, *p* = 0.027, $\eta_{p}^{2}$ = 0.19; see **Figure 4C**]. Simple effects *t*-tests revealed that high ambiguity led to a significant reduction in JoAs relative to low ambiguity in no turbulence trials (mean difference = 3.28%, *SD* = 6.86, *t*_(23)_ = 2.35, *p* = 0.028, *d*_z_ = 0.48), but there was no effect of ambiguity in turbulence trials [mean difference = -0.71%, *SD* = 6.78, *t*_(23)_ = -0.52, *p* = 0.61, *d*_z_ = -0.11]. Turbulence led to a significant reduction in JoAs in both ambiguity conditions [low ambiguity: mean difference = 39.90%, *SD* = 17.26, *t*_(23)_ = 11.33, *p* < 0.001, *d*_z_ = 2.31; high ambiguity: mean difference = 35.91%, *SD* = 17.20, *t*_(23)_ = 10.23, *p* < 0.001, *d*_z_ = 2.09].

Importantly, in this experiment, the ambiguity manipulation – varying the proportions of more and less ambiguous items – was relatively subtle. Therefore, participants’ sensitivity to the difference between ambiguity conditions, and its effect on JoAs, could depend on the strategy participants employed while playing the game. Some might try to maximize their hits by catching many items, while risking a larger number of false alarms. Others might be very wary of false alarms, and thus focus on catching the least ambiguous items, even though they reduced their number of hits. One hypothesis would be that participants making risky choices would be more affected by the ambiguity manipulation, because they would make more uncertain decisions, i.e., deciding to catch an item that they were uncertain about, with higher ambiguity. On the other hand, more careful participants could be more affected by the manipulation because they would be more sensitive to the overall increased uncertainty in the decision process, i.e., deciding which items to catch, in the high ambiguity condition. These strategies were captured as the average response bias (Green and Swets, 1966) of each participant, with larger (positive) values indicating more careful responding.

Therefore, in addition to assessing whether the effect of ambiguity on JoAs, could be accounted for by changes in JoPs, as in previous experiments, we assessed whether it was related to participants’ average bias. For this, we added average bias, and its interaction with ambiguity, to the previously used model. As before, results showed a positive relation between JoPs and JoAs (*b* = 0.05, *t*_(22.56)_ = 7.34, *p* < 0.001, 95% CI = [0.04, 0.07]), and a significant effect of turbulence (*b* = -0.35, *t*_(23.38)_ = -9.83, *p* < 0.001, 95% CI = [-0.42, -0.28]; see **Figure 4D** and Supplementary Table 2). Consistent with the ANOVA results, the main effect of ambiguity was not significant (*b* = -0.02, *t*_(196.38)_ = -1.26, *p* = 0.21, 95% CI = [-0.05, 0.01]). However, the ambiguity by turbulence interaction, which was significant in the ANOVA, was no longer significant in this model (*b* = 0.03, *t*_(390.40)_ = 1.60, *p* = 0.11, 95% CI = [-0.01, 0.08]). Participants’ average bias was not significantly related to JoAs overall (*b* = 0.17, *t*_(22.82)_ = 1.48, *p* = 0.15, 95% CI = [-0.05, 0.43]), but more importantly, there was a significant interaction between ambiguity and average bias (*b* = -0.16, *t*_(194.37)_ = -2.60, *p* = 0.01, 95% CI = [-0.29, -0.03]). Model predictions, displayed in **Figure 4E**, showed that participants with a larger average bias, that is, with more conservative responding, showed a larger ambiguity effect. [For exploratory analyses of how response bias was affected by our experimental manipulations, at the within-participant level and across experiments, see Supplementary Analyses in Data Sheet 1.]

### Discussion

The present experiment investigated the effect of uncertainty in action selection on the sense of agency by manipulating stimulus ambiguity. In Experiment 1, once stimuli were identified amidst the noise mask, it was clear whether they were a target or a distractor. In contrast, items were easy to detect here, but there was uncertainty about the categorization of highly ambiguous stimuli. Results showed that objective and subjective measures of performance were reduced by greater stimulus ambiguity, and by the turbulence manipulation. Average JoAs were also reduced by turbulence, but were only affected by stimulus ambiguity in the condition without turbulence. That is, when the cursor accurately followed the mouse’s movements, greater stimulus ambiguity led to a decrease in JoAs, but there was no difference in JoAs when the mouse and cursor movements were discrepant.

In line with the previous experiment, these findings support the proposal that metacognitive signals about action selection can influence the sense of agency (Chambon et al., 2014b). They further extend previous research by showing that uncertainty about the correct response to a stimulus can lead to a loss of agency. Interestingly, stimulus ambiguity reduces confidence judgements (Boldt and Yeung, 2015), suggesting there may be overlap in the signals that inform both types of metacognitive judgements.

The discrepancy between intended and observed movements, induced by mouse turbulence, again had a larger effect on JoAs than the visual manipulation (in this case, ambiguity), and, in fact, abolished stimulus ambiguity effects. A similar interaction between masking and turbulence was found in Experiment 1. The turbulence manipulation was attenuated in the present experiment, and its effect on performance was more similar in size to the effect of ambiguity, relative to the difference between the effects of turbulence and masking in Experiment 1. However, the increased overall difficulty of this task, even in the “low ambiguity” condition, may have led to weaker ambiguity effects on metacognitive judgements, as the difference between conditions was less clear.

Furthermore, this experiment allowed participants to use different strategies in playing the game. Some might risk making uncertain decisions, by catching the more ambiguous items, even though they could be distractors. Others might avoid making such risky decisions, and limit themselves to catching the less ambiguous items. To account for these differences in strategies across participants, we included a measure of the average response bias of participants when modeling JoAs. On the one hand, stimulus ambiguity might particularly affect the sense of agency when one makes more uncertain decisions. In this case, participants who made more uncertain decisions, i.e., those with a lower response bias, would presumably be more sensitive to the ambiguity manipulation. At the same time, these risky participants might have been less concerned with whether they caught a target or distractor. On the other hand, stimulus ambiguity might have affected the sense of agency by making the task more difficult, since there were fewer easy-to-categorize items in the high ambiguity condition. Then, participants who restricted themselves to making more certain decisions, i.e., those with a higher response bias, would have been more affected by the ambiguity manipulation.

Modeling results showed that the interaction between ambiguity and turbulence found for average JoAs may be partly explained by differences in JoPs. Additionally, there was a significant interaction between ambiguity and participants’ average response bias, which was independent of JoPs. This showed that stimulus ambiguity had a larger effect on JoAs in participants who had a more conservative criterion for responding, or a more positive bias, relative to less conservative participants. Participants who restricted themselves to catching those stimuli that were unambiguously identifiable as targets were most affected by the ambiguity manipulation. In the high ambiguity condition there were fewer unambiguous items, therefore the task was more difficult.

This could also reflect an overall effect of action frequency on sense of agency: conservative participants who required unambiguous evidence to identify targets for action would make relatively fewer actions during the game, especially in the high ambiguity condition. They would therefore feel a reduced sense of agency, compared to less conservative participants who made more actions. Interestingly, the interaction pattern observed in **Figure 4E** shows little difference in JoAs across participants for the high ambiguity condition. Instead, it suggests that it was more conservative participants who showed an increase in JoAs in the low ambiguity condition. Perhaps these participants felt especially certain of their decisions in this condition, whereas liberal participants who made more risky decisions always felt highly uncertain about their decisions, regardless of the ambiguity condition.

These results suggest that participants’ JoAs were especially sensitive to the contextual effect of stimulus ambiguity on task difficulty, i.e., whether it was easier or harder to identify targets. It remains unclear how uncertainty in a specific decision influences the sense of agency, since the dynamic nature of the game allowed participants to avoid making more uncertain decisions. Yet, in everyday life, there are many situations in which one cannot avoid making a decision, even when one is uncertain. Thus, it could still be hypothesized that, under conditions where avoiding a decision is not possible, uncertainty about the action could influence the sense of agency.

## Experiment 3

This experiment tested the effect of response conflict on the sense of agency, by adding task-relevant flankers to target and distractor stimuli.

### Materials and Methods

#### Participants

Procedures were as described above. Twenty-three new Columbia University or Barnard College students (18 female, mean age = 23.87, *SD* = 4.87) volunteered to participate for course credit, and gave written informed consent. Three participants were left-handed, one was ambidextrous, and the remaining were right-handed. One participant was excluded due to extremely low JoPs and JoAs (>2 SD below the mean, across conditions), and very low performance in the condition with no disruptions (full congruency and no turbulence: *d’* was 2 SDs below the mean).

#### Apparatus

The main apparatus was as in Experiment 2, except for the targets and distractors. Groups of three letters, consisting of Ms and Cs, were presented in white on a black background. Groups with a target letter in the central position were defined as target groups, and groups with a distractor letter in the central position were defined as distractor groups. Each trial started with six target groups and six distractor groups.

#### Design and Procedure

The design and procedure were as in previous experiments, except for the following changes. In the present experiment, Ms and Cs were presented as targets and distractors (counterbalanced). To manipulate action selection, flanker letters were added to the target and distractor items, in order to induce response conflict when flankers were incongruent with the middle letter (e.g., CMC). Items always consisted of three-letter groups, but participants were instructed to focus on the middle letter. Only the central letter counted as a target or distractor, and participants had to touch the central letter with the mouse cursor (white box) in order to catch it. Touching only the outer letters did not count as a catch. Therefore, the flanker letters should be ignored. As before, participants played the game for 30 s, and then gave JoAs and JoPs. Instructions were adapted for the present manipulation.

**FIGURE 5:**
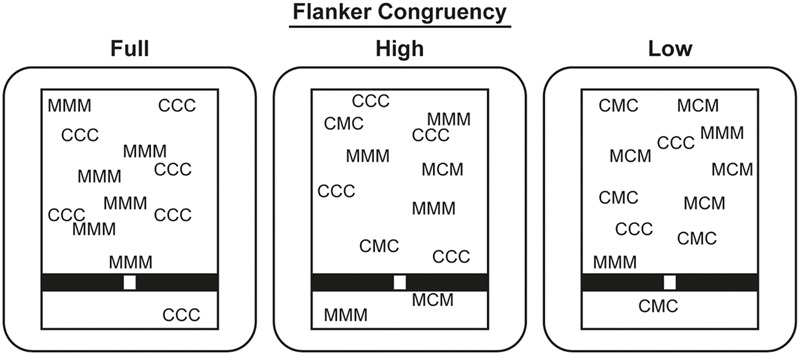
**Schematic of stimuli in Experiment 3.** Stimulus size has been adapted, and colors have been inverted.

Flanker congruency was manipulated across three conditions (see **Figure 5**). In the full congruency condition, all items were surrounded by congruent flankers (i.e., always MMM and CCC). In the high congruency condition, two-thirds of the items were congruent, but the other third was incongruent (i.e., CMC and MCM). Finally, in the low congruency condition, only one-third of the items was congruent, and the other two-thirds were incongruent. Additionally, turbulence was manipulated across trials, as in Experiment 2. This 3 × 2 factorial design resulted in six conditions, which were quasi-randomized across six blocks, as before.

During training, participants first started by playing a game with full flanker congruency, and practiced JoAs and JoPs. Once confident with this condition, they were introduced to the incongruently flanked items, and played another practice game with high flanker congruency, followed by JoAs and JoPs. They were given the chance to practice further, if required.

#### Data Analysis

Mean *d’*, JoPs and JoAs were submitted to repeated measures ANOVAs with the factors flanker congruency (full vs. high vs. low) and turbulence (no turbulence vs. turbulence). Planned comparisons were used to probe the main effect of congruency. Additionally, JoAs were modeled by the experimental factors and by JoPs, as in Experiment 1, except for the coding of congruency. The three-level factor congruency resulted in two contrasts, with full congruency as a baseline condition (i.e., full vs. high and full vs. low).

### Results

**FIGURE 6:**
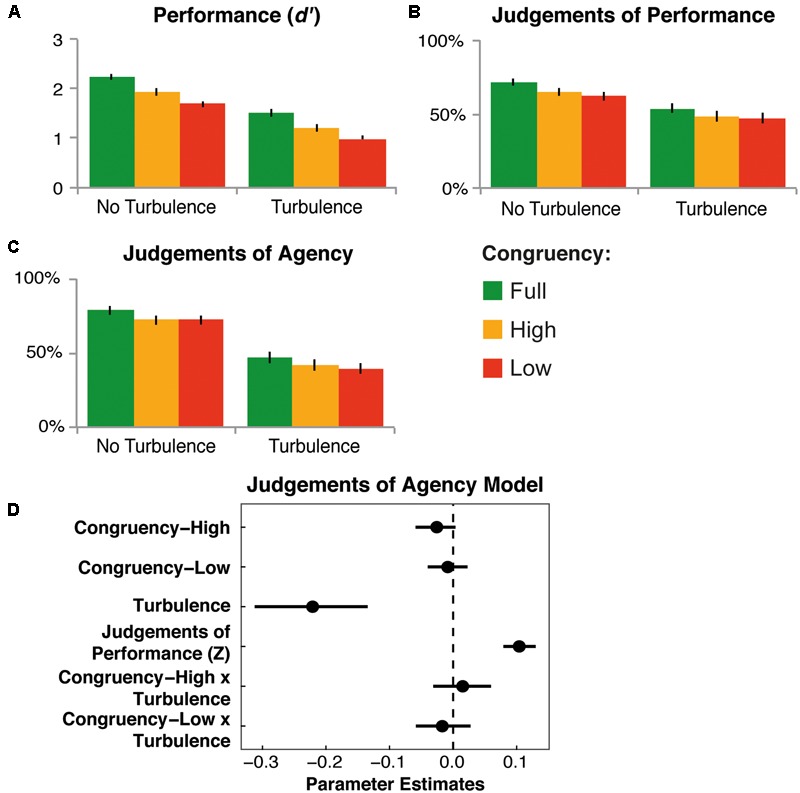
**Results of Experiment 3.** Effects of flanker congruency and turbulence on mean *d’* **(A)**, JoPs **(B)**, and JoAs **(C)**. No Turb, no turbulence; Turb, turbulence. Error bars show the standard error of the mean. **(D)** Parameter estimates, with bootstrapped 95% confidence intervals, for modeling JoAs by the independent variables and by JoPs (*Z*-score, within participants).

Discrimination between targets and distractors (*d’*) was significantly affected by flanker congruency [*F*_(2,42)_ = 105.41, *p* < 0.001, $\eta_{p}^{2}$ = 0.83; see **Figure 6A**]. High congruency led to a significant reduction in performance, relative to full congruency (full – high: mean = 0.30, *SD* = 0.14), and low congruency led to a further significant reduction relative to high (high – low: mean = 0.23, *SD* = 0.19; all comparisons *p* < 0.001). Turbulence led to a significant reduction in performance, relative to no turbulence [mean difference = 0.72, *SD* = 0.21, *F*_(1,22)_ = 253.70, *p* < 0.001, $\eta_{p}^{2}$ = 0.92]. There was no significant interaction between the factors [*F*_(2,42)_ = 0.23, *p* = 0.80, $\eta_{p}^{2}$ = 0.011].

Analyses of JoPs showed a significant effect of flanker congruency [*F*_(2,42)_ = 31.36, *p* = 0.001, $\eta_{p}^{2}$ = 0.60; see **Figure 6B**]. Relative to full congruency, high congruency led to a significant reduction in JoPs (full – high: mean = 5.87%, *SD* = 5.16, *p* < 0.001), and there was a further reduction in JoPs for low congruency, relative to high (high – low: mean = 2.27%, *SD* = 4.99, *p* = 0.042). Turbulence led to a significant reduction in JoPs, relative to no turbulence [mean difference = 16.61%, *SD* = 11.14, *F*_(1,22)_ = 48.49, *p* < 0.001, $\eta_{p}^{2}$ = 0.70]. The interaction between the factors was not significant [*F*_(2,42)_ = 0.86, *p* = 0.39, $\eta_{p}^{2}$ = 0.039, Greenhouse-Geisser corrected].

Flanker congruency also influenced JoAs significantly [*F*_(2,42)_ = 19.91, *p* < 0.001, $\eta_{p}^{2}$ = 0.49; see **Figure 6C**]. JoAs were significantly lower in the high and low congruency conditions, relative to full congruency (full – high: mean = 5.39%, *SD* = 4.80; full – low: mean = 6.66%, *SD* = 4.69; *p*s < 0.001). JoAs did not differ between high and low congruency conditions (high – low: mean = 1.27%, *SD* = 6.12; *p* = 0.33). Turbulence led to a reduction in JoAs, relative to no turbulence [mean difference = 31.48%, *SD* = 21.00, *F*_(1,22)_ = 49.19, *p* < 0.001, $\eta_{p}^{2}$ = 0.70]. The interaction between the factors was not significant [*F*_(2,42)_ = 0.71, *p* = 0.47, $\eta_{p}^{2}$ = 0.033, Greenhouse-Geisser corrected].

Modeling JoAs with JoPs as a covariate (see **Figure 6D** and Supplementary Table 3) again showed a significant positive relation between JoPs and JoAs (*b* = 0.10, *t*_(21.15)_ = 7.90, *p* < 0.001, 95% CI = [0.08, 0.13]), and turbulence remained a significant predictor of JoAs (*b* = -0.22, *t*_(20.71)_ = -4.98, *p* < 0.001, 95% CI = [-0.31, -0.13]). The contrast between full and high congruency was not a significant predictor of JoAs (*b* = -0.03, *t*_(60.60)_ = -1.65, *p* = 0.10, 95% CI = [-0.06, 0.00]), and neither was the full vs. low congruency contrast (*b* = -0.01, *t*_(57.39)_ = -0.55, *p* = 0.58, 95% CI = [-0.04, 0.02]). This shows that the effects of flanker congruency on JoAs could be largely explained by changes in JoPs. There were no significant interactions between the congruency contrasts and turbulence (full vs. high × turbulence: *b* = 0.01, *t*_(53.87)_ = 0.69, *p* = 0.49, 95% CI = [-0.03, 0.06]; full vs. low × turbulence: *b* = -0.02, *t*_(111.32)_ = -0.83, *p* = 0.41, 95% CI = [-0.06, 0.03]).

### Discussion

In Experiment 3, action selection fluency was manipulated by varying the congruency between flankers and targets or distractors. Incongruent flankers were used to induce response conflict, as one might be mistakenly drawn toward a distractor or away from a target. Results showed that, indeed, flanker congruency affected both objective and subjective measures of performance. Additionally, and consistent with previous findings (Sidarus and Haggard, 2016), flanker congruency influenced metacognitive judgements of agency. Parametrically reducing the proportion of congruent flankers led to a gradual reduction in performance. JoPs also showed a gradual reduction in performance, but with a larger reduction when comparing full and high congruency, relative to comparing high and low congruency. This confirms that the presence of incongruent flankers led to an impairment in performance. Moreover, it suggests that, the presence of incongruent flankers was more salient than their proportion at a metacognitive level.

In fact, the results showed that average JoAs were only sensitive to the presence or absence of incongruent flankers. Relative to full congruency, both high and low congruency conditions led to a significant reduction in JoAs, but there was no difference between high and low congruency. The mere presence of some incongruent flankers was sufficient to disrupt the sense of agency, independently of the proportion of incongruent to congruent flankers, and thus independently of the precise degree of task difficulty they caused. Previous studies already showed that the influence of response conflict on the sense of agency was independent of RTs, which is typically considered an index of difficulty of each particular trial (Chambon and Haggard, 2012; Sidarus and Haggard, 2016). Furthermore, previous studies using a similar game have shown that, while JoAs are sensitive to the presence of discrepancy between the mouse’s movements and visual feedback, they are not particularly sensitive to the degree of that discrepancy (Metcalfe et al., 2010, 2012; Zalla et al., 2015). Thus, sense of agency is highly sensitive to any disruption of intentional action, but does not track in detail the *degree* of disruption.

These results are consistent with the view that a positive sense of agency may be a “default” state, and it is when the normal flow from intention to action to outcome is disrupted that the sense of agency is reduced (Chambon et al., 2014b). Relative to the default state (in this case, the full congruency/no turbulence condition), any disruption is quite salient, whereas the degree of disruption may be less important. When any part of the stimulus-action-outcome processing chain is disrupted, the sense of agency may be reduced. However, details about the disruption that informs metacognitive judgements of agency can be somewhat unspecific, not only about the locus of the disruption, but also about the degree of the disruption. Indeed, fluency/conflict signals are known to often be vague and unspecific in content, and can affect other metacognitive judgements (Winkielman et al., 2015).

As predicted, turbulence once again led to a large reduction in JoAs, which was greater than the effect of introducing incongruent flankers. Unlike Experiments 1 and 2, in which manipulations of action selection interacted with turbulence, there was no significant congruency by turbulence interaction for JoAs. As can be seen in **Figure 6**, there was a clear additive effect of the two manipulations on performance, and to some extent on JoPs, but the pattern was less clear for JoAs. Nevertheless, turbulence seemed to be a more important cue to agency.

Finally, modeling of JoAs revealed that they were only predicted significantly by JoPs and turbulence condition. This suggests that changes in JoPs accounted for a large part of the congruency effects on JoAs. While this might seem to imply that flanker congruency, and thus that response conflict does not have an effect on the sense of agency independently of performance monitoring, this conclusion would be premature. Previous studies have shown that response conflict leads to a reduction in the sense of agency, independently of monitoring the external consequences of the action (e.g., the appearance of a colored circle; cf. Chambon et al., 2014b; Sidarus and Haggard, 2016), as well as monitoring performance in terms of RTs (Chambon and Haggard, 2012; Sidarus and Haggard, 2016).

Whereas the aforementioned studies mostly measured the sense of agency after each fluent or dysfluent action, the present experiment involved a dynamic interaction with varying proportions of congruent/incongruent flankers, and thus assessed a more global experience of fluency vs. conflict, after the participant made several different actions in the game. The present experiment shows, as “proof of concept,” that an accumulated experience of conflict can influence the metacognition of agency. This is consistent with studies which obtained agency ratings at the end of a block, and showed that experiences of fluency or conflict became associated with specific action outcomes (Wenke et al., 2010; Sidarus and Haggard, 2016).

Nonetheless, further research is needed to explore a possible dissociation between the effects of conflict on JoAs and JoPs. For example, the use of three congruency levels here could have led to an overall reduction in the congruency effect on JoAs, relative to an experiment comparing only full and high congruency conditions. The frequent exposure to incongruent flankers, due to the inclusion of both high and low congruency conditions, could have weakened the required stimulus-response association (i.e., catch Ms and avoid Os). This would, in turn, have weakened the conflict triggered by incongruent flankers, and thus reduced their impact on JoAs. We may therefore speculate that reinforcing the standard stimulus-response mapping by increasing the prevalence of congruent flankers could yield a greater effect of response conflict on the sense of agency.

## General Discussion

Previous psychological research emphasized how sense of agency depends on a process of comparing the predicted and actual consequences of actions. This tradition of research has been largely based on explicit judgements of whether a specific outcome did or did not match one’s own actions. Thus, our focus on action selection represents a novel theoretical departure for sense of agency research (cf. Chambon et al., 2014b). The present research goes beyond previous studies in three important ways. First, we investigated the contribution of action selection processes to the sense of agency, in contrast to the traditional emphasis on outcomes. Second, we investigated the sense of agency while acting in a dynamic environment requiring continuous coordination between visual input and motor output. This allowed us to study sense of agency in a rich, ecological context, which emphasized fluent control of performance, as opposed to discrete matching of events. Finally, previous work on the role of action selection to the sense of agency had so far only employed response conflict tasks (Chambon et al., 2014b; Sidarus and Haggard, 2016). We extended this work by using different manipulations of action selection, across three experiments: stimulus processing fluency, stimulus ambiguity, and response conflict.

We consistently found that disrupting action selection processes led to reductions in JoAs. These novel manipulations, applied in a dynamic environment, reveal the robustness and generalisability of the effects of action selection fluency on agency. Action selection necessarily precedes action itself, and also precedes action outcomes. Thus, our results show that prospective cues, based on action selection, contribute to sense of agency.

Our view complements the dominant emphasis on the role of retrospective monitoring of outcomes in sense of agency. In particular, we showed how prospective cues are integrated with retrospective cues to agency, based on proximal outcomes. For this, we combined manipulations of action selection with the classical discrepancy between movements and visual feedback used to disrupt retrospective processing. Discrepancy led to a loss of agency, in accordance with previous studies (Metcalfe and Greene, 2007; Farrer et al., 2008; Metcalfe et al., 2010, 2013). Across all experiments, discrepancy remained the predominant cue to agency. The effects of action selection on sense of agency were dramatically reduced when discrepancy was also present. That is, when the cursor accurately followed the mouse’s movements, action selection made a larger contribution to JoAs than when the cursor was perturbed. This interaction was most marked when action selection processing was manipulated through visual processing fluency and stimulus ambiguity factors.

Unlike other agency studies (e.g., Haggard et al., 2002), the continuous and rich context of our task allowed us to measure performance, i.e., achieving the goal of catching Xs. We also asked participants to make judgements of their own performance. Discrepant visual feedback affected performance and JoPs more strongly than did our manipulations of action selection. These strong effects of discrepancy on performance could explain why effects of action selection on JoAs were small compared to effects of discrepancy. This could also explain the weaker effects of action selection on JoAs when visual feedback was discrepant, as discrepancy already led to floor effects on JoAs.

The present findings appear to contrast, however, with a previous study that showed action selection fluency had a larger effect on the sense of agency when outcomes violated expectations (Sidarus et al., 2013). However, that study required participants to judge *distal* outcomes of actions (colored circles), whereas we manipulated *proximal* outcomes here. Furthermore, as those distal outcomes were mostly predictable (67% contingency), the occasional violation of expectations was not a reliable indicator of loss of agency. Under such circumstances, relying on internal cues to agency would compensate for the expected uncertainty (Yu and Dayan, 2005) of the external environment. On the other hand, the present study found that discrepancy between the mouse and cursor movements (a proximal outcome) did reliably indicate a loss of agency, as participants had objectively less control over the game. While the manipulations of action selection made the game more or less difficult to play, they did not affect participants’ objective ability to control the game. Previous studies showed that sense of agency is more sensitive to disruption of the relation between movement and proximal outcomes than to disruption of the relation between movement and distal outcomes (Metcalfe et al., 2013). The present results emphasize the predominance of proximal outcomes, by showing that proximal outcomes may overshadow effects of action selection on sense of agency.

Finally, as manipulations of action selection can also affect task performance, we tested whether effects on JoAs could be partially accounted for by changes in subjective evaluations of performance (i.e., JoPs). As seen in previous studies (e.g., Metcalfe and Greene, 2007; Metcalfe et al., 2013), JoPs were positively related to JoAs, and the effects of discrepant visual feedback on JoAs could not be accounted for by JoPs across all three experiments. Regarding action selection, in Experiment 1, visual processing fluency had an effect on JoAs that was independent of changes in JoPs. In Experiment 2, we additionally considered the behavioral strategies of participants in playing the game, and found that the effect of stimulus ambiguity on more cautious, conservative participants was not fully accounted for by JoPs. However, in Experiment 3, the effects of response conflict on JoAs appeared to be largely explained by JoPs. Notably, the weaker effects of the action selection manipulations on JoAs in Experiments 2 and 3 may have allowed for a greater influence of JoPs.

Our major conclusion, therefore, is that action *selection* processes inform the sense of agency, at least when actions and their proximal outcomes are consistent. This view highlights the role of *prospective* contributions to sense of agency, in contrast with previous research in the field, which emphasizes the retrospective matching of outcomes with actions, or with intentions. Our results suggest that even before we act, the process of constructing a feeling of agency has already begun. This prospective sense of agency is thought to serve as an advance predictor of successful action, and to bridge the interval between action and outcome (Chambon et al., 2014b). The results described here confirm the role of this cue in the sense of agency, while consistently showing that the actual statistical relation between action and (proximal) outcome remains the most important cue. This relative weighting between selection processes and proximal outcomes may be important in estimating control.

A prospective sense of agency based on action selection is, of course, not a veridical perception of the actual degree of control. The true degree of control a participant has in the task refers to the relation between their motor action and the movement of the cursor on the screen. That is, the processes that lead participants to *choose how to act* are independent of the effect that their action, once they actually make it, will have in the task. Thus, the fluency or difficulty of action selection gives only an *estimate* of true statistical control. In general, this estimate is a reliable heuristic for control: when one clearly and easily knows which action to make to achieve a goal, this will generally be because the relation between actions and goals is stable, and the goal can be successfully achieved.

The view that sense of agency is an illusion has recently been popular in psychology (Alloy and Abramson, 1979; Wegner and Wheatley, 1999). When outcomes match expectations, people typically have a strong sense of agency, and thus may be fooled into feeling in control, even when their action has not in fact produced the outcome, or when they have made no action at all (Wegner and Wheatley, 1999; Wegner et al., 2004). However, this view fails to account for why we may sometimes achieve our desired goal, and yet still feel like we are not fully in control. For example, suppose a person who has never played darts before hits the bull’s eye at the first throw. Even though the desired goal was obtained (hitting the bull’s eye), that person will likely not feel fully in control of that outcome. Rather, the agent might partially attribute the outcome to some “beginner’s luck,” and thus experience a reduced sense of agency. The action performed was not precisely and fluently selected, given the agent’s lack of expertise in the game. We suggest that the dysfluency in action selection serves here as an important signal to the agent of a lack of control over the task. Although the desired outcome was obtained *this time*, the lack of expertise with the task, and the resulting dysfluency in action selection, means the agent feels she is *unlikely* to achieve the same outcome *next time*. Therefore, signals of fluency or dysfluency, and the varying sense of agency that accompanies them, can serve to guide future behavior. For example, someone who feels that their success reflects “beginner’s luck” may still feel a need for further learning. In order to optimally estimate control, the sense of agency must integrate various cues, and their relative contribution, or weights, must be adaptively changed based on which cues are more reliable indicators of control, in a particular context (Moore and Fletcher, 2012; Synofzik et al., 2013). In our “beginner’s luck” example, the beginner may give a low weighting to their (successful) outcome and a high weighting to the dysfluency of their action selection. Thus, expertise with a given context may moderate how much the action- vs. outcome-related cues affect the sense of agency.

**FIGURE 7:**
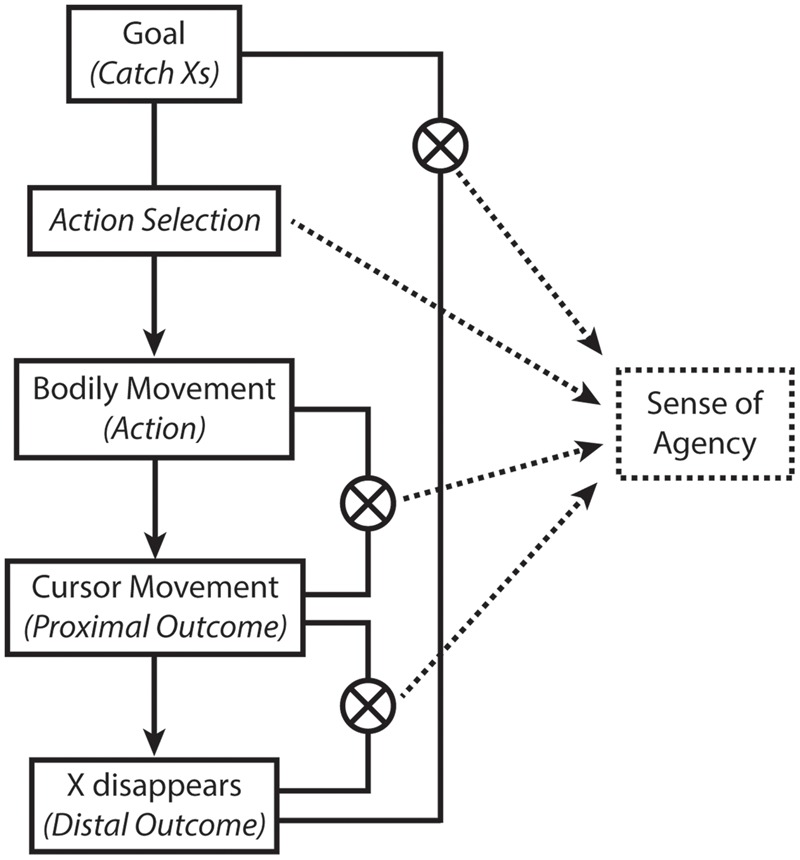
**An integrative and hierarchical model of the sense of agency.** Various cues to the sense of agency are integrated, which may arise from different levels of hierarchies of intentions (converting goals into actions) and outcomes (proximal vs. distal).

Goal-directed action is underpinned by a hierarchical structure of intentions, actions, and outcomes. To achieve an overarching goal (distal intentions, in the terminology of Pacherie, 2008), specific actions (proximal intentions) must be chosen, and converted into motor plans (motor intentions). These various levels of the hierarchy must be monitored, and behavior adjusted as needed, in order to achieve the goal. We present a schematic model of how this could occur within our task (see **Figure 7**). The participant’s goal is to catch Xs. For this, they have to move the cursor on the screen to the vertical location of targets. Action selection processes serve to convert the high level goal into specific manual movements of the computer mouse. The (dys)fluency of this selection process serves as a first input into the sense of agency, with dysfluency signals leading to a reduction in the sense of agency. Next, the movements of the cursor on the screen (proximal outcomes) must be compared to the predicted movements (based on the motor commands). This will provide a second input to agency. For example, any mismatch due to the experimental manipulation of “turbulence” will reduce the sense of agency. Finally, once the cursor is moved to the correct location to intercept the X, the X is expected to disappear – this is the distal outcome, or goal of the task. If, however, the X did not disappear when touched (cf. Metcalfe et al., 2013), this would induce a mismatch between the expected outcome of intercepting the X, implying a failure to achieve the (distal) goal of catching Xs. These distal mismatch signals would also provide a third input to the sense of agency. Monitoring the different levels of this hierarchy is important both for determining how the various signals should influence the sense of agency, as well as for determining whether/how one’s behavior should be adjusted in order to achieve the desired goal.

Clearly, further research is needed to better understand the mechanisms and computations underlying cue integration in the sense of agency. Here, we focused on the role of prospective cues, and testing the robustness and generalizability of previous findings. However, it remains unclear whether the different disruptions of action selection used here would constitute the same cue, as output of a common action monitoring system, or whether they might constitute different inputs to the sense of agency. Suggestively, a recent study into the neural correlates of prospective cues to agency (Sidarus et al., 2017), which induced response conflict, found a correlation between the correct-related negativity (CRN) component of action-locked event-related potentials (ERPs) and judgements of agency. A similar relation has been reported between the CRN and confidence judgements in tasks which varied processing fluency (Scheffers and Coles, 2000) and discrimination difficulty (Boldt and Yeung, 2015). Future studies could take advantage of neural measures, such as ERPs, to further investigate potential commonalities between disruptions of action selection and their influence on the sense of agency. Additionally, combining implicit and explicit measures of agency would help clarify the similarities/differences in cue integration for different aspects of the experience of agency. Finally, other contextual factors, and inter-individual variability, which were not explored here, could influence the sense of agency, or cue integration. It should be noted that gender was not fully balanced across all of the present studies, and the potential impact of gender on manipulations of the sense of agency remains unclear.

## Conclusion

Our work suggests that the sense of agency is based on a continuous integration of several different cues relevant to the relation between actions and distal outcomes. We show that the proximal outcomes (here, moving the visual cursor) that constitute the means toward achieving a distal goal (here, intercepting Xs) have a strong influence on the sense of agency. Interestingly, the ease or difficulty of selecting which action to make also consistently had some influence on the sense of agency. Thus, the human sense of agency is clearly integrative and synthetic. Sense of agency is not simply the perception of a match between one discrete action event and one corresponding discrete outcome event. Rather, it reflects integration of several different classes of signal. Our key contribution has been to demonstrate the contribution of internal and metacognitive signals related to action selection, in addition to signals related to bodily movement and external outcome.

## Author Contributions

All authors contributed to the development and design of the studies. NS developed the software, conducted the experiments, and analyzed the data, with MV’s help. NS drafted the manuscript, and MV, JM, and PH provided critical revisions. All authors approved the final version of the manuscript for submission.

## Conflict of Interest Statement

The authors declare that the research was conducted in the absence of any commercial or financial relationships that could be construed as a potential conflict of interest.
