# Supplementary material for: Investigating the Prospective Sense of Agency: Effects of Processing Fluency, Stimulus Ambiguity, and Response Conflict
Source: Front Psychol. 2017 Apr 13;8:545. doi: 10.3389/fpsyg.2017.00545 (PMC5389984; doi:10.3389/fpsyg.2017.00545)
Supplement: Supplementary file 1 [file Table_1.pdf]

Supplementary Table 1. JoAs by factors and JoPs (within participants Z) model for Experiment 1: parameter estimates, with bootstrapped 95% confidence intervals. * *Based on the Satterthwaite approximation (Kuznetsova, Brockhoff, & Christensen, 2015)*

						C.I.	
	Estimate	S.E.	*t*	df*	*p**	2.5 %	97.5 %
(Intercept)	0.70	0.03	24.68	21.23	< 0.001	0.65	0.77
Masking	-0.05	0.02	-2.98	21.52	0.007	-0.08	-0.02
Turbulence	-0.34	0.04	-8.80	20.27	< 0.001	-0.41	-0.26
JoPs (Z)	0.12	0.02	6.39	22.56	< 0.001	0.09	0.16
Masking x Turb	0.03	0.02	1.48	51.90	0.14	-0.01	0.08
