# Supplementary material for: Investigating the Prospective Sense of Agency: Effects of Processing Fluency, Stimulus Ambiguity, and Response Conflict
Source: Front Psychol. 2017 Apr 13;8:545. doi: 10.3389/fpsyg.2017.00545 (PMC5389984; doi:10.3389/fpsyg.2017.00545)
Supplement: Supplementary file 2 [file Table_2.pdf]

Supplementary Table 2. JoAs by factors and JoPs (within participants Z) and participants' average bias (centered, between participants) model for Experiment 2: parameter estimates, with bootstrapped 95% confidence intervals. * *Based on the Satterthwaite approximation (Kuznetsova, Brockhoff, & Christensen, 2015)*

						C.I.	
	Estimate	S.E.	t	df*	p*	2.5 %	97.5 %
(Intercept)	0.71	0.03	23.82	22.60	< 0.001	0.65	0.77
Ambiguity	-0.02	0.02	-1.26	196.40	0.21	-0.05	0.01
Turbulence	-0.35	0.04	-9.83	23.40	< 0.001	-0.42	-0.28
JoPs (Z)	0.05	0.01	7.34	22.60	< 0.001	0.04	0.07
Average Bias	0.17	0.12	1.48	22.80	0.15	-0.05	0.43
Ambiguity x Turb	0.03	0.02	1.60	390.40	0.11	-0.01	0.08
Ambiguity x Average Bias	-0.16	0.06	-2.60	194.40	0.01	-0.29	-0.03
