# Supplementary material for: Investigating the Prospective Sense of Agency: Effects of Processing Fluency, Stimulus Ambiguity, and Response Conflict
Source: Front Psychol. 2017 Apr 13;8:545. doi: 10.3389/fpsyg.2017.00545 (PMC5389984; doi:10.3389/fpsyg.2017.00545)
Supplement: Supplementary file 3 [file Table_3.pdf]

Supplementary Table 3. JoAs by factors and JoPs (within participants Z) model for Experiment 3: parameter estimates, with bootstrapped 95% confidence intervals. * *Based on the Satterthwaite approximation (Kuznetsova, Brockhoff, & Christensen, 2015)*

						C.I.	
	Estimate	S.E.	t	df*	p*	2.5 %	97.5 %
(Intercept)	0.71	0.03	23.77	21.06	< 0.001	0.64	0.77
Congruency-High	-0.03	0.02	-1.65	60.60	0.10	-0.06	0.00
Congruency-Low	-0.01	0.02	-0.55	57.39	0.58	-0.04	0.02
Turbulence	-0.22	0.04	-4.98	20.71	< 0.001	-0.31	-0.13
JoPs (Z)	0.10	0.01	7.90	21.15	< 0.001	0.08	0.13
Cong-High x Turb	0.01	0.02	0.69	53.87	0.49	-0.03	0.06
Cong-Low x Turb	-0.02	0.02	-0.83	111.32	0.41	-0.06	0.03
