# Supplementary material for: Investigating the Prospective Sense of Agency: Effects of Processing Fluency, Stimulus Ambiguity, and Response Conflict
Source: Front Psychol. 2017 Apr 13;8:545. doi: 10.3389/fpsyg.2017.00545 (PMC5389984; doi:10.3389/fpsyg.2017.00545)
Supplement: Supplementary file 4 [file Data_Sheet_1.pdf]

Supplementary Analyses

In Experiment 2, we assessed whether participants' strategy in playing the game might influence their sensitivity to our ambiguity manipulation, by computing an average response bias (*c*) and considering variability at the between-subjects level. In an exploratory analysis, we assessed whether response bias might also vary consistently based on our experimental manipulations – at a within-subjects level – by performing repeated-measures ANOVA on response bias, for each experiment.

In Experiment 1, we found only a marginal effect of noise ($F_{(1, 22)}$=4.21, p=.052), with a tendency for a higher bias (i.e. more conservative responding) in the masking condition. In Experiment 2, we found only a significant effect of turbulence ($F_{(1, 23)}$=5.74, p=.025), with higher bias in the turbulence condition. In Experiment 3, we found only a significant effect of flanker congruency ($F_{(2, 42)}$=5.70, p=.006), with a linear increase in bias with the reduction in the proportion of congruent/incongruent items (i.e. more incongruent items). There were no other significant effects (p>.1).

These results suggest that participants tended to respond more carefully, i.e. were more conservative, when action selection was disrupted, i.e. when it was harder to decide where to move one's cursor to, in Experiments 1 & 3. The absence of a within-subject main effect of ambiguity in Experiment 2 is consistent with our hypothesis, and finding, that variability at the between-subject level in how participants' strategy in playing the game overall (reflected in their average response bias) was associated with variability in participants' sensitivity to the ambiguity manipulation.

The effect of turbulence on response bias was not robust across experiments. We might speculate that, in Experiments 1 & 3, the mere presence of turbulence did not lead participants to refrain from seeking out targets, even though it may have impaired their ability to place the cursor under the *correct* item (hence the resulting cost in performance, i.e. in *d'*). Perhaps the effect of turbulence on response bias seen only in Experiment 2 is due to the fact that the task of discriminating targets and foils was already very difficult, so the difficulty added by the turbulence manipulation led participants to be even more cautious in their responses. Intriguingly, the amount of noise introduced in the turbulence condition in Experiments 2 and 3 was smaller than the one used in Experiment 1, supporting the relevant of the discrimination task itself on response bias.
